# Natural History of Type II Autosomal Dominant Osteopetrosis: A Single Center Retrospective Study

**DOI:** 10.3389/fendo.2022.819641

**Published:** 2022-03-17

**Authors:** Ziyuan Wang, Xiang Li, Ya Wang, Wenzhen Fu, Yujuan Liu, Zhenlin Zhang, Chun Wang

**Affiliations:** Shanghai Clinical Research Center of Bone Disease, Department of Osteoporosis and Bone Diseases, Shanghai Jiao Tong University Affiliated Sixth People’s Hospital, Shanghai, China

**Keywords:** *CLCN7*, osteopetrosis, follow-up, genotype, phenotype

## Abstract

**Background:**

Autosomal dominant osteopetrosis II (ADO II, MIM166600) is a sclerosing bone disorder caused by *CLCN7* mutation. The main clinical characteristics include minor trauma-related fracture and hip osteoarthritis, whereas cranial nerve palsy and bone marrow failure rarely develop. Although it is generally believed that ADO II has a relatively benign course, the natural course of the disease in Chinese patients remains unclear.

**Materials and Methods:**

Thirty-six patients diagnosed with ADO II in Shanghai Jiao Tong University Affiliated Sixth People’s Hospital from 2008 to 2021 were studied retrospectively. Among them, 15 patients were followed for an average of 6.3 years (1-14 years).

**Results:**

In this study, minor trauma-related fractures of the limb were the most typical clinical manifestations. Visual loss (1/36) and bone marrow failure (2/36), was rare in this study. The condition of ADO II seems to be stable in most patients. There were no correlations between markedly elevated bone mineral density (BMD) and minor trauma-related fractures. In total, 21 diseases causing mutations were detected. Among them, the mutation c.2299C>T (p.Arg767Trp) was the most common (16.67%), and mutation c.937G>A [p.(Glu313Lys)] was associated with severe fractures, haematological defects and cranial palsy.

**Conclusions:**

Minor trauma-related fracture is the most typical clinical manifestation of ADO II and always occurs in. The mutation c.2299C>T (p.Arg767Trp) is in general a relatively common variant, while the mutation c.937G>A [p.(Glu313Lys)] seems to be associated with severe phenotype. In our study, ADO II seems to remain stable over time.

## Introduction

Autosomal dominant osteopetrosis II (ADO II) is a genetic bone disorder caused by mutation in the *CLCN7* (chloride channel 7) gene, which encodes CLC-7, a Cl^-^/H^+^ exchanger that provides the chloride conductance required for efficient proton pumping in the osteoclast ruffled membrane (1). Mutation in *CLCN7* may disrupt acidification of the osteoclast resorption lacunae, resulting in impaired bone degradation (2).

ADO II is a disorder that shows large clinical heterogeneity (3). The main clinical characteristics of ADO II comprise minor trauma-related fracture, hip osteoarthritis and osteomyelitis of the mandible and other bones (4). In severe cases, the sclerotic bone compressing the cranial nerve and bone marrow cavity causes visual loss and bone marrow failure (3). Classical radiographic signs include “sandwich vertebrae” and “bone-within-bone” in the vertebrae and pelvis (5, 6).

To date, the correlation between the phenotype and genotype and natural history of ADO II clinical manifestations in China needs further study. Here, we study 36 subjects with *CLCN7* heterozygous mutations and explore the clinical manifestations and disease development over time.

## Study Subjects and Methods

### ADO II Patients and Definition of the ADO II Phenotype

For this study, data of patients who were diagnosed with ADO II between 2008 and 2021 in Shanghai Jiao Tong Affiliated Sixth’s People’s Hospital was collected (7–11). All subjects who participated in the study signed informed consent documents prior to enrolment. We collected clinical data through patient interviews and physical examinations, medical records, and/or self-reported responses on a questionnaire completed by all subjects. The diagnosis of ADOII depends on clinical manifestations, typical imaging examinations and *CLCN7* gene mutation (12).

### Assessment and Definition of Clinical Manifestation

Assessment of the main clinical manifestations included fracture, discomfort of joints and even osteoarthritis, osteomyelitis, cranial nerve palsy such as visual or aural loss and abnormal complete blood counts. Minor trauma-related fracture was primarily ascertained by the patient**’**s medical history. Fractures are divided into three degrees depending on their distribution and number in individuals: no fracture, fracture and severe fracture. Severe fracture means 10 or more fractures of any type and/or more than one hip/femur fracture (13). Osteoarthritis was considered present if the patients had clinical manifestations and exhibited changes on imaging according to the criterion of American College of Rheumatology and the radiological grading system of Kellgren and Lawrence (14–16). Osteomyelitis was considered present if (1) a patient was treated with antibiotics due to bone infection and (2) there was a history of gum breakdown or dental extraction with osteonecrosis. Visual loss was considered only if patients had blindness or decreased vision with well-documented impairment. Haematological defects were defined as extramedullary haemopoiesis or necessary blood transfusion due to decreased bone marrow haematopoiesis.

### Biochemical and Radiographic Examination

Serum levels of alkaline phosphatase (ALP), calcium (Ca), phosphate (P), beta cross-linked carboxy-terminal telopeptide of type I collagen (β-CTX), 25-hydroxyvitamin D (25OHD), intact parathyroid hormone (PTH), creatine kinase (CK) and the CK MB isoenzyme (CK-MB) were measured using an automated Roche electrochemiluminescence system (E170; Roche Diagnostic GmbH, Mannheim, Germany). All serum biochemical parameters were measured in the central clinical laboratory of Shanghai Jiao Tong University Affiliated Sixth People’s Hospital. The results of individual X-rays of the thoracic and lumbar vertebrae, hips and skulls were obtained.

### Bone Densitometry

Bone mineral density (BMD) was accomplished with dual X-ray absorptiometry (DXA) (GE Lunar Corp.,Madison, WI, United States). All DXA scans were conducted by a trained specialist. The BMD results were converted to Z scores by comparison to age- and sex-matched normal Chinese children or adults (17).

### Longitudinal Data

In the current study, 15 patients were followed up for 1 to 14 years (average 6.3 years). The follow-up included changes in clinical manifestations (fractures, discomfort of joints even osteoarthritis and others), BMD, radiological examinations and laboratory examinations.

### Statistical Analyses

Statistical analyses were performed in SPSS for Windows version 25.0 (SPSS Inc., Chicago, IL, United States). Continuous variables with a normal distribution were compared using Student’s t test, and the results are expressed as the mean ± standard deviation. The Spearman correlation test was used to analyse the relationship between fractures and BMD measurements. P value < 0.05 was considered statistically significant.

## Results

### Demographic and Clinical Characteristics

**Figure 1:**
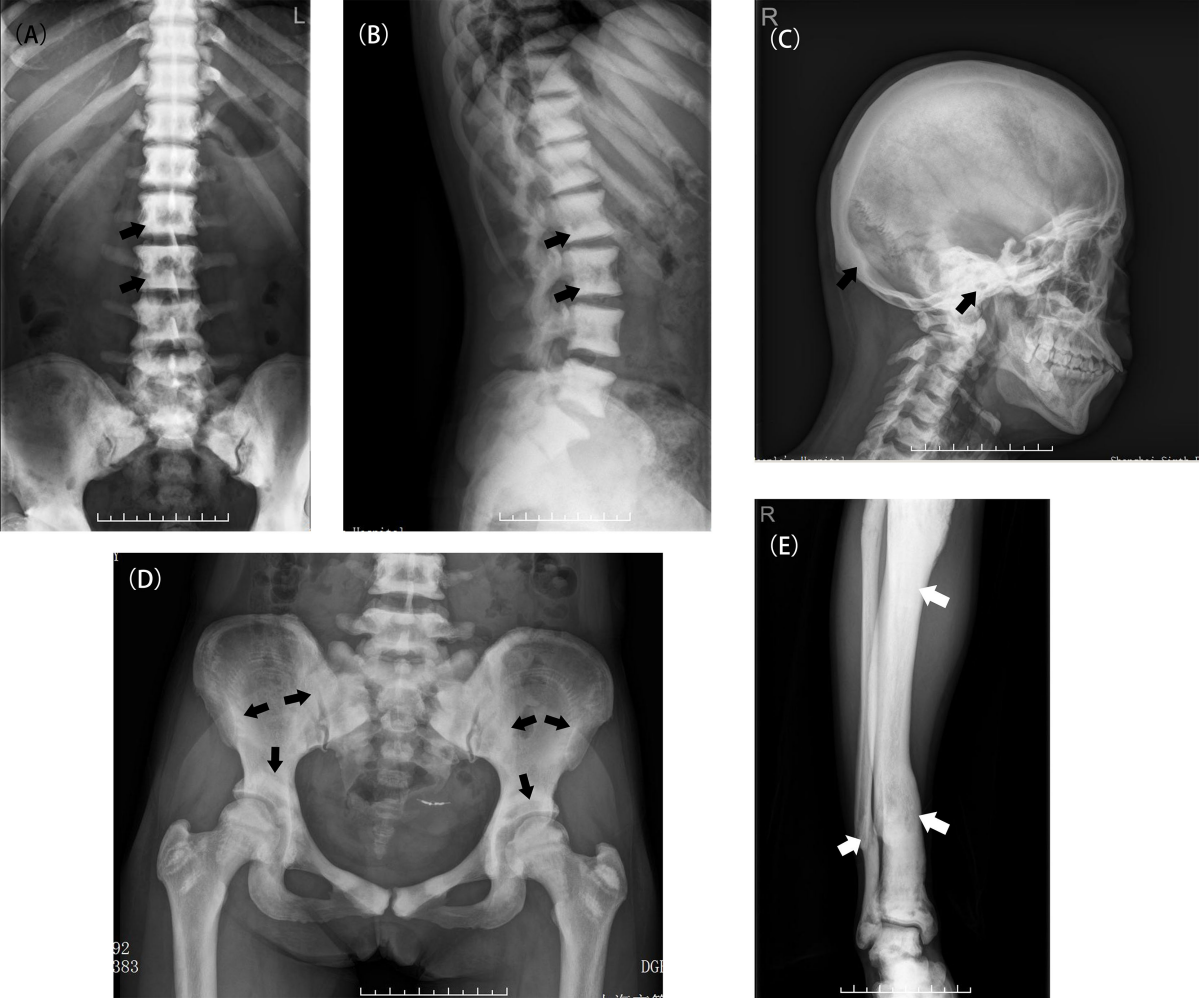
Typical X-ray examinations of ADO II **(A, B)** A patient (P2) with classic radiograph findings including diffuse osteosclerosis and the appearance of a rugger-jersey spine (black arrow). **(C)** A patient (P2) with a sclerotic skull, especially in the basis cranii (black arrow). **(D)** The typical appearance of “bone within bone” in the Patient 5 (black arrow). **(E)** A deformed tibia due to multiple fractures in the Patient 2(white arrow).

In total, 36 patients in 28 unrelated families aged from 8 months to 75 years were enrolled in this study. The penetrance was 69.23% in this study. **Table 1** as a supplementary showed the specific information about the patients and unaffected carrier. The median age at diagnosis was 19 years. The youngest patient (Patient 30, P30) had varus deformity of the left hip. The oldest patient (P29) had only one fracture history (clavicle fracture), at 9 years old, and had knee osteoarthritis for 40 years. Among the 36 patients, 26 (75.6%) were diagnosed with osteopetrosis when they visited a hospital because of fracture, osteoarthritis or other symptoms. The remaining 10 patients (24.4%) had no or mild symptoms and were only occasionally diagnosed with osteopetrosis. All patients displayed typical radiographic ‘‘sandwich vertebrae’’ and ‘‘bone-within-bone’’ changes (**Figure 1**).

**Table 1:** *CLCN7* mutation in ADO II families.

Family	Family member	Status	Relationship	Nucleotide change
F1	P1	Patient	Proband’s sister	c.2299C>T
	P2	Patient	P1’s son	c.2299C>T
	P3	Patient	Proband	c.2299C>T
	P4	Patient	Proband’s son	c.2299C>T
F2	P5	Patient	Proband	c.2299C>T
	Carrier 1 (C1)	Healthy carrier	Proband’s father	c.2299C>T
	C2	Healthy carrier	Proband’s son	c.2299C>T
F3	P6	Patient	Proband	c.2299C>T
F4	P7	Patient	Proband	c.296A>G
	P8	Patient	Proband’s father	c.296A>G
F5	P9	Patient	Proband	c.296A>G
F6	P10	Patient	Proband	c.296A>G
	C3	Healthy carrier	Proband’s father	c.296A>G
F7	P11	Patient	Proband	c.857G>A
	P12	Patient	Proband’s mother	c.857G>A
F8	P13	Patient	Proband	c.857G>A
	P14	Patient	Proband’s father	c.857G>A
	C4	Healthy carrier	Proband’s sister	c.857G>A
F9	P15	Patient	Proband	c.937G>A
	P16	Patient	Proband’s sister	c.937G>A
	C5	Healthy carrier	Proband’s mother	c.937G>A
F10	P17	Patient	Proband	c.937G>A
	C6	Healthy carrier	Proband’s mother	c.937G>A
F11	P18	Patient	Proband	c.2236T>G
	P19	Patient	Proband’s twin sister	c.2236T>G
F12	P20	Patient	Proband	28974C>T
F13	P21	Patient	Proband	28974C>T
F14	P22	Patient	Proband	c.746C>T
F15	P23	Patient	Proband	c.856C>T
	C7	Healthy carrier	Proband’s father	c.856C>T
F16	P24	Patient	Proband	c.865G>C
	C8	Healthy carrier	Proband’s mother	c.865G>C
	C9	Healthy carrier	Proband’s sister	c.865G>C
F17	P25	Patient	Proband	19852C>T
F18	P26	Patient	Proband	c.896C>T
	C10	Healthy carrier	Proband’s mother	c.896C>T
F19	P27	Patient	Proband	20247C>G
F20	P28	Patient	Proband	c.953T>C
	C11	Healthy carrier	Proband’s father	c.953T>C
F21	P29	Patient	Proband	c.955T>A
F22	P30	Patient	Proband	c.1625C>T
F23	P31	Patient	Proband	28968G>C
	C12	Healthy carrier	Proband’s father	28968G>C
	C13	Healthy carrier	Proband’s sister	28968G>C
F24	P32	Patient	Proband	c.2258C>G
F25	P33	Patient	Proband	c.2284C>T
	C14	Healthy carrier	Proband’s father	c.2284C>T
	C15	Healthy carrier	Proband’s sister	c.2284C>T
F26	P34	Patient	Proband	2392-/G
	C16	Healthy carrier	Proband’s father	2392-/G
F27	P35	Patient	Proband	c.2332-2A>G
F28	P36	Patient	Proband	c.2250+1G>A

Seventeen patients had a history of fracture, and the total number of fractures was 40. Three persons had severe fracture: two from multiple femur fractures and the other from a total of nine fractures, including two in the femur. **Table 2** showed the fracture sites, treatment and recovery outcomes of patients.

**Table 2:** Sites, treatment and healing of patients’ fracture.

Patient	Site	Treatment	Heal
P1	Left femoral neck	Total hip arthroplasty	/
P2	Right radioulna (3 times), right tibiofibula	Conservative treatment	Healed
P3	Right humerus	NA	NA
P4	Right dorsum of foot	Conservative treatment	Healed
P7	Right clavicle, left femoral neck	NA	Healed
P10	Left tibia, radius	Conservative treatment	Healed
P14	Left proximal femur, left femoral shaft	Internal fixation	Non-healed
P15	Right radius, right little finger, bilateral radioulna (2 times)	Conservative treatment	Healed
P16	NA	NA	NA
P17	Left femur (2 times), right femur	NA	Healed
P18	Left distal radius	Conservative treatment	Healed
P20	Right femur	Internal fixation	Non-healed
P21	Left tibia	Conservative treatment	Healed
P25	Right tibia, right ankle, right middle finger, right radioulna	NA	NA
P27	NA	NA	NA
P28	Wrist	Conservative treatment	Healed
P29	Clavicle fracture	NA	NA
P31	Right twelfth rib, proximal phalanx, right proximal femur, left femur (total 9 times)	NA	NA
P34	Left elbow joint, left ankle, right ankle, coccygeal vertebra	Conservative treatment	Healed
P35	Rib	Conservative treatment	Healed

NA, Not available.

Seventeen patients (47.2%) felt discomfort of the joint, and 5 of them showed X-ray changes. Affected joints included vertebral joints (9/17), the hip (4/17), the knee (3/17) and the shoulder joint (1/17). **Table 3** showed the details of clinical manifestations, radiology and treatment of patients with osteoarthritis. Other clinical manifestations included anaemia (5/36), haematological defects (1/36), visual loss (1/36) and osteomyelitis (1/36). **Table 4** summarizes the clinical manifestations of all patients at the first visit.

**Table 3:** Clinical manifestations, radiology and treatment of patients with osteoarthritis.

Patient	Clinical manifestation	Radiology and K-L grade	Treatment
P1	Pain in the right hip several years and aggravation on ambulation	Acetabular osteophytes and femoral and acetabular sclerosis, Grade 2	NSAIDs if necessary
P2	Pain in the right hip for 10 years, pain while at rest and aggravation on ambulation	Deformity of femoral head, joint space narrowing even slight joint fusion, femoral and acetabular sclerosis, Grade 3	NSAIDs, rehabilitation therapy
P14	Pain in bilateral hip for several years, pain while at rest	Deformity of femoral head, joint space narrowing and femoral and acetabular sclerosis, Grade 3	NA
P29	Knee pain for 40 years and aggravation on ambulation	Osteophytes and subchondral sclerosis, Grade 2	NSAIDs
P35	Shoulder intermittent pain for several years without traumatic history, aggravation with exercise	humeral head osteophytes, the subchondral sclerosis in the humerus and glenoid, Grade 2	NSAIDs and reduction of exercise

NA, Not available.

**Table 4:** Demographic and clinical characteristics of 36 ADO II patients at their first visit.

No.	Family	Age of first visit (years)	Amino acid change	Fractures	Joint discomfort or osteoarthritis	Others
P1(11)	F1	51	p.Arg767Trp	1	Hip osteoarthritis	Hb 89g/L, hypochromic microcytic anemia
P2(8)	F1	32	p.Arg767Trp	4	Hip osteoarthritis	No	
P3(8)	F1	34	p.Arg767Trp	1	Lumbodorsal pain	No	
P4(8)	F1	6	p.Arg767Trp	1	No	No	
P5(8)	F2	33	p.Arg767Trp	0	Lumbodorsal pain	No	
P6(9)	F3	27	p.Arg767Trp	0	Neck discomfort	Hb 78g/L, normochromic normocytic anemia	
P7(9)	F4	5	p.(Tyr99Cys)	2	No	No	
P8(9)	F4	29	p.(Tyr99Cys)	0	No	No	
P9	F5	15	p.(Tyr99Cys)	0	Knee discomfort	No	
P10(10)	F6	10	p.(Tyr99Cys)	2	No	Recurrent influenza	
P11	F7	3.7	p.(Arg286Gln)	0	No	No	
P12	F7	43	p.(Arg286Gln)	0	Knee discomfort	No	
P13	F8	13	p.(Arg286Gln)	0	Hip pain	No	
P14	F8	44	p.(Arg286Gln)	2	Hip osteoarthritis	Tinnitus	
P15(8)	F9	11	p.(Glu313Lys)	2	No	No	
P16(8)	F9	21	p.(Glu313Lys)	>1	No	Hb 112g/L, hypochromic microcytic anemia and mandibular osteomyelitis	
P17(10)	F10	32	p.(Glu313Lys)	3	No	Hb 71g/L, hypochromic microcytic anemia, PLT 83×10^9^/L, splenomegaly, visual loss and tinnitus	
P18(10)	F11	42	p.(Tyr746Asp)	0	No	No	
P19(10)	F11	42	p.(Tyr746Asp)	0	No	No	
P20(8)	F12	31	p.(Arg743Trp)	0	Lumbodorsal pain	Hb 100g/L, and nerve deafness	
P21(8)	F13	31	p.(Arg743Trp)	1	Lumbodorsal pain	No	
P22(11)	F14	18	p.(Pro249Leu)	0	Lumbodorsal pain	No	
P23(10)	F15	16	p.(Arg286Trp)	0	Lumbodorsal pain	Lumbar spina bifida	
P24(9)	F16	35	p.(Val289Leu)	0	No	No	
P25(8)	F17	8	p.(Ser290Phe)	4	No	No	
P26(7)	F18	12	p.(Ala299Val)	0	No	Pain in the left foot	
P27(8)	F19	10	p.(Ala316Gly)	1	No	No	
P28(11)	F20	5	p.(Phe318Ser)	0	No	No	
P29(7)	F21	75	p.(Trp319Arg)	1	Knee osteoarthritis	No	
P30(9)	F22	0.8	p.(Ala542Val)	0	No	Varus deformity of the left hip	
P31(8)	F23	11	p.(Gly741Arg)	9	No	No	
P32(11)	F24	61	p.(Ser753Trp)	0	No	No	
P33(11)	F25	34	p.(Arg762Trp)	0	Lumbodorsal pain	No	
P34(8)	F26	17	p.(Glu789GlyfsX129)	3	Lumbodorsal pain	No	
P35(10)	F27	28	c.2332-2A>G	1	Omarthritis	No	
P36	F28	8	c.2250+1G>A	0	No	Hb 107g/L, iron-deficiency anemia and pain in the left foot	

Hb, hemoglobin.

The normal ranges in adults are Hb 115-150g/L, MCV 82-100fl, MCH 27-34pg, MCHC 316-354g/L, PLT 125-350×10^9^/L.

### Assessment of BMD

**Figure 2:**
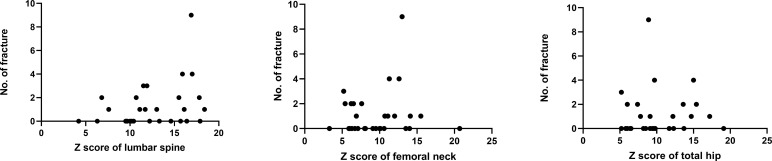
Relationship between BMD Z scores and fractures.

BMD was measured in 31 patients. P11 and P30 were too young to take a BMD examination. The medical history and X-ray results of P4, P14 and P19 were provided by their families so they lacked of BMD measurements. P17 suffered bilateral femur fractures so she can’t take the BMD examination of hip. The mean Z scores of the lumbar spine, femoral neck and total hip of the rest were 12.7 ± 3.8 SD (n=31; range: 4.2-18.4), 9.6 ± 3.8 SD (n=30; range: 3.3-20.7) and 10.0 ± 3.8 SD (n=30; range: 5.2-19.1), respectively. There was no statistical significance in mean Z score at the lumbar spine, femoral neck or total hip between patients up to 18 years old and patients older than 18 years. There was no statistical significance between the number of fractures and Z score at lumbar and hip sites (p=0.140, p=0.712 and p=0.417), but **Figure 2** displays a mild trend in the way that higher Z scores are associated with higher number of fractures.

### Biochemical Parameters of ADO II Patients

Serum levels of bone turnover markers Ca, P and ALP in all patients were within the normal range (Serum Ca: 2.08-2.60mmol/L, Serum P: 0.80-1.60mmol/L, ALP: 18-112U/L). However, eight patients had elevated PTH levels, and 16 patients had low 25(OH)D levels (normal range of PTH: 15-65pg/ml, 25(OH)D >20ng/mL). Fifteen patients showed elevated levels of CK, with almost all patients having high CK-MB levels.

### Follow-Up

Of the 36 patients, 15 were followed up for 1 to 14 years. Five patients experienced new fractures. Three of them (P15, P18, P28) had fractures in the radius or ulna, one (P34) had a coccygeal vertebral fracture, and one (P20) had a femoral fracture. The patient P20 suffered from a femoral fracture at the age of 34 and treated with internal fixation. P34 had a coccygeal vertebral fracture at the age of 29 and took a conservative treatment. P15, P18 and P28 took a conservative treatment. **Table 3** showed the fracture sites, treatment and recovery outcomes of all patients. Joint discomfort in four patients was relieved without intaking pain killers such as Non-steroidal anti-inflammatory drugs (NSAIDs). Two patients with osteoarthritis had no significant exacerbation of symptoms, and no patient experienced osteomyelitis during follow-up. One patient with severe fractures, visual loss and haematological defects (P17) was in stable condition and without new fractures and haematopoietic failure, such as frequent infection or haemorrhage. One patient (P20) felt fatigue during follow-up, and complete blood test revealed mild anaemia, thrombocytopaenia and splenomegaly. Only one patient (P16) died of pneumonia at the age of 31, 10 years after diagnosis.

Except for one patient (P20), BMD remained stable for several years of follow-up. **Tables 5**, **6** presents the clinical characteristics and changes in BMD during follow-up.

**Table 5:** Changes in clinical manifestations during follow-up.

Patient	Time of follow-up (years)	Fracture		Osteoarthritis		Visual loss		Hearing impairment		Leukocytopenia		Anemia		Thrombocytopenia		Hepato-splenomegaly	
		Baseline	Follow-up	Baseline	Follow-up	Baseline	Follow-up	Baseline	Follow-up	Baseline	Follow-up	Baseline	Follow-up	Baseline	Follow-up	Baseline	Follow-up
P1	5	1	No	Osphyarthrosis	Osphyarthrosis	No	No	No	No	No	NA	Moderate	NA	No	NA	No	NA
P5	14	No	No	Lumbodorsal pain	Relief	No	No	No	No	No	No	No	No	No	No	No	No
P9	1	No	No	Knee discomfort	Relief	No	No	No	No	No	No	No	No	No	No	NA	NA
P13	1	No	No	Hip pain	Relief	No	No	No	No	No	NA	No	NA	No	NA	NA	NA
P14	1	2	No	Osphyarthrosis	Osphyarthrosis	No	No	No	No	NA	NA	NA	NA	NA	NA	NA	NA
P15	8	2	2	No	No	No	No	No	No	No	No	No	No	No	No	No	No
P17	4	3	No	No	No	Yes	Yes	Tinnitus	No	No	NA	Moderate	NA	Yes	NA	Splenomegaly	NA
P18	4	No	1	No	No	No	No	No	No	No	NA	No	NA	No	NA	NA	NA
P20	12	No	1	Lumbodorsal pain	Knee discomfort	No	No	Nerve deafness	No	No	No	Mild	Mild	No	Yes	NA	Splenomegaly
P21	13	1	No	Lumbodorsal pain	Knee discomfort	No	No	No	No	No	No	No	No	No	No	NA	NA
P22	6	No	No	Lumbodorsal pain	Relief	No	No	No	No	No	NA	No	NA	No	NA	NA	NA
P28	5	No	1	No	No	No	No	No	No	No	NA	No	NA	No	NA	No	NA
P34	14	3	1	Lumbodorsal pain	Relief	No	No	No	No	No	No	No	No	No	No	No	No
P35	4	1	No	Shoulder osteoarthritis	Shoulder osteoarthritis	No	No	No	No	No	No	No	No	No	No	NA	NA
P36	1	No	No	No	No	No	No	No	No	No	No	Mild	Mild	No	No	Hepatomegaly	No

NA, Not available.

**Table 6:** Changes of BMD Z scores during follow-up.

Patient	Time of follow-up (years)	L1-4		Neck		Total hip	
		Baseline	Follow-up	Baseline	Follow-up	Baseline	Follow-up
P1	5	18.4	NA	15.5	NA	17.2	NA
P5	14	9.5	13.7	10.6	10.3	9.6	9.1
P9	1	14.6	15.4	10.1	10.1	9.1	9.2
P13	1	9.7	NA	6.7	NA	6.5	NA
P14	1	NA	NA	NA	NA	NA	NA
P15	8	17.8	16.2	6.5	7.4	13.6	8.9
P17	4	11.5	NA	NA	NA	NA	NA
P18	4	9.6	NA	6	NA	6	NA
P20	12	16.4	25.8	20.7	19.7	19.1	17.9
P21	13	13	NA	10.7	NA	12.2	NA
P22	6	16.4	NA	8.1	NA	9.4	NA
P28	6	17.9	NA	14	NA	12.3	NA
P34	14	11.9	8.9	5.2	5.4	5.2	4.4
P35	4	11.1	10.8	11.1	9.6	9.1	8.1
P36	1	15.7	16.2	13.5	15.5	13.7	14.9

NA, Not available.

### Genotype-Phenotype Correlations

Twenty-one disease-causing mutations in *CLCN7* were detected in 36 patients. Four common mutations, c.2299C>T (p.Arg767Trp), c.296A>G [p.(Tyr99Cys)], c.857G>A [p.(Arg286Gln)] and c.937G>A [p.(Glu313Lys)], were present in 16.2%, 10.8%, 10.8% and 8.1% of the patients, respectively. **Table 7** shows details regarding *CLCN7* mutations in the 36 patients.

**Table 7:** *CLCN7* mutations in ADO II patients.

Number	Nucleotide change	Amino acid change	Mutation type	Cases	Frequency
1	c.2299C>T	p.Arg767Trp	Missense	6	16.2%
2	c.296A>G	p.(Tyr99Cys)	Missense	4	10.8%
3	c.857G>A	p.(Arg286Gln)	Missense	4	10.8%
4	c.937G>A	p.(Glu313Lys)^a^	Missense	3	8.1%
5	c.2236T>G	p.(Tyr746Asp)^a^	Missense	2	5.4%
6	28974C>T	p.(Arg743Trp)^a^	Missense	2	5.4%
7	c.746C>T	p.(Pro249Leu)	Missense	1	2.7%
8	c.856C>T	p.(Arg286Trp)	Missense	1	2.7%
9	c.865G>C	p.(Val289Leu)^a^	Missense	1	2.7%
10	19852C>T	p.(Ser290Phe)^a^	Missense	1	2.7%
11	c.896C>T	p.(Ala299Val)^a^	Missense	1	2.7%
12	20247C>G	p.(Ala316Gly)^a^	Missense	1	2.7%
13	c.953T>C	p.(Phe318Ser)^a^	Missense	1	2.7%
14	c.955T>A	p.(Trp319Arg)^a^	Missense	1	2.7%
15	c.1625C>T	p.(Ala542Val)^a^	Missense	1	2.7%
16	28968G>C	p.(Gly741Arg) ^a^	Missense	1	2.7%
17	c.2258C>G	p.(Ser753Trp)^a^	Missense	1	2.7%
18	c.2284C>T	p.(Arg762Trp)	Missense	1	2.7%
19	c.2332-2A>G^a^		Intron splice	1	2.7%
20	c.2250+1G>A		Intron splice	1	2.7%
21	2392-/G	p.(Glu789GlyfsX129)^a^	Frameshift	1	2.7%

a indicates a novel mutation we has reported from 2008 to 2021.

GenBank accession number of the CLCN7 variant cDNA: NM_001287.6.

Almost all 6 patients (P1-P3, P5-P6) with p.Arg767Trp mutations had discomfort of the joint and even osteoarthritis. Four of them experienced fractures, one with multiple fractures. Three patients (P15-P17) with the p.(Glu313Lys) mutation had severe clinical manifestations. All three patients had multiple fractures; one (P17) had severe fractures, visual loss and haematological defects. The other patient (P16) had mild anaemia and mandibular osteomyelitis and died at the age of 31 years. Two patients (P7, P10) harbouring the p.(Tyr99Cys) mutation had fractures; in contrast, patients (P11-13) carrying the p.(Arg286Gln) mutation had mild symptoms. **Table 8** shows the relationship between mutation site and clinical characteristics and auxiliary examinations.

**Table 8:** Clinical characteristics and auxiliary examinations in association with four common mutations.

Mutation	No. of fractures	Osteoarthritis	L1-4Z score	FNZ score	THZ score	ALP (U/L)	CK (U/L)	CK-MB (U/L)
p.Arg767Trp	7	2	13.6 ± 4.8	11.1 ± 3.2^*^	11.9 ± 4.0^*^	112.5 ± 112	208.4 ± 101.3	247.5 ± 120.9
p.(Tyr99Cys)	4	0	9.9 ± 3.3	7.2 ± 2.1	8.1 ± 3.1	135.8 ± 50.3	278.3 ± 183.6	329 ± 318.4
p.(Arg286Gln)	2	1	8.8 ± 5.9	5.0 ± 1.5^*^	6.2 ± 1.1^*^	217 ± 195.8	320.7 ± 275.3	242.5 ± 212.9
p.(Glu313Lys)	>8	0	12.8 ± 3.0	7.5 ± 0.1	12.2 ± 3.2	116.7 ± 15.6	180.3 ± 112.6	276.5 ± 350

The normal ranges of serum ALP, CK and CK-MB are 11–112 U/L, 21–190 U/L, and 0–25 U/L, respectively.

The normal ranges of serum ALP and CK in patients from 2 to 18 years old are 58–400 U/L and 10–90 U/L, respectively.

*L1-4, Lumbar 1-4, FN, Femoral neck; TH, Total hip.

^*^: Z scores at the femoral neck and total hip between p.Arg767Trp and p.(Arg286Gln) mutations are significantly different.

**Figure 3:**
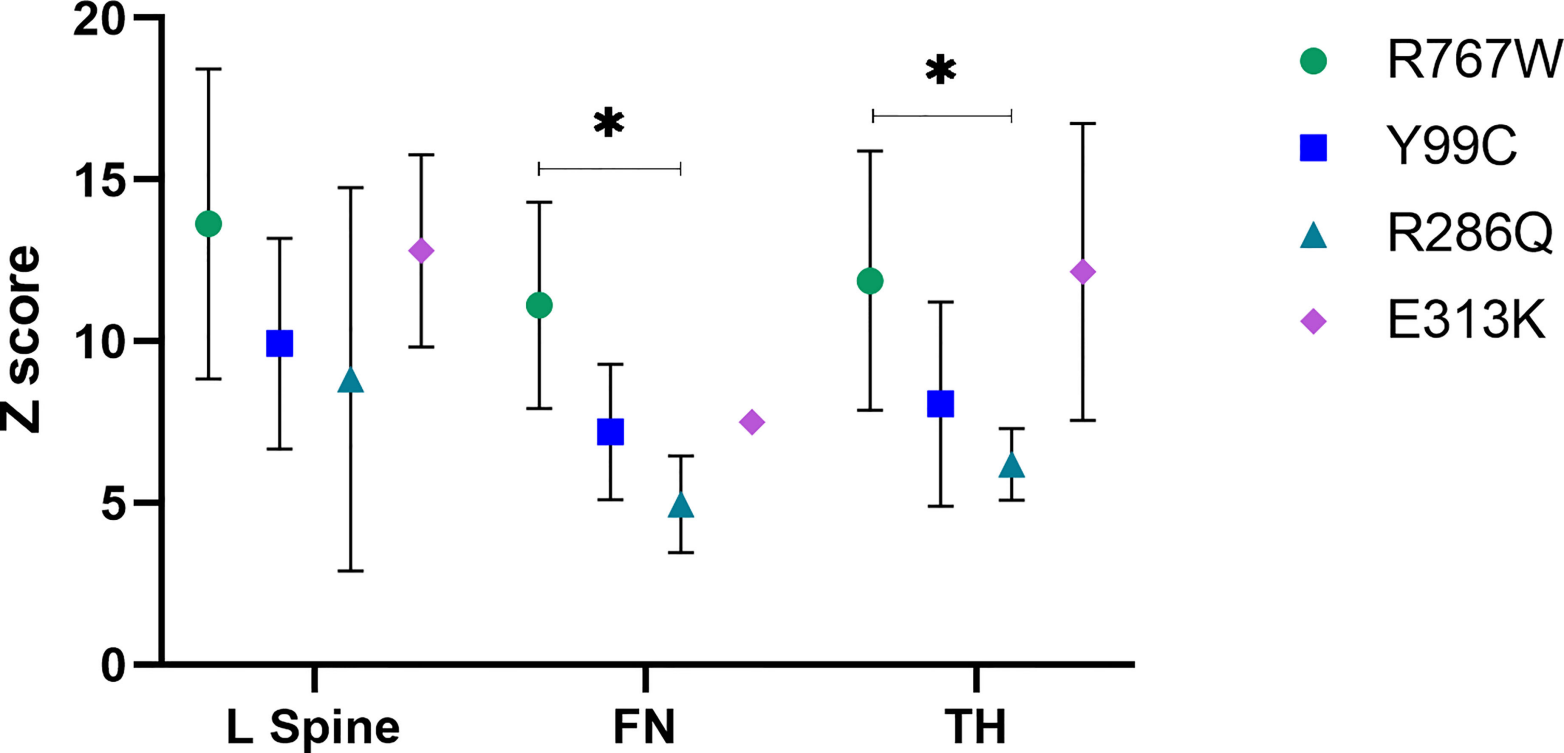
Central DXA measurements in ADO > subjects with four mutations and the relationship between mutations and BMD at lumbar spine and hip sites. *means p<0.05. L spine, Lumbar spine; FN, femoral neck; TH, Total hip.

Compared with p.(Arg286Gln) mutation, patients with p.Arg767Trp mutant exhibited significantly elevated Z scores of BMD at the femoral neck and total hip. **Figure 3** illustrates the relationship between mutation and BMD at lumbar spine and hip sites. Biochemical parameters, including CK and CK-MB, were elevated in those with the four common mutations. **Table 7** summarizes the clinical characteristics, BMD measurements and biochemical parameters of patients carrying the four common mutations.

## Discussion

ADO II is a sclerotic bone disorder with high genetic heterogeneity. ADO II is often considered a benign disorder; however, the authors of some studies consider that “benign osteopetrosis” is a misnomer (18, 19). Fractures are the most typical manifestation of the disease. Fracture of the long bones of extremities are frequent and caused by decreased resistance to torsional forces (18).

In 2007, Waguespack and colleagues (13) evaluated 94 ADO II patients and summarized their clinical manifestations. The rate of fracture was 84% in their study, and these authors found that adults were more likely than children to experience fracture. This over 30-year follow-up study showed that the symptoms of both ADO II patients and healthy carriers got worse during follow-up. Therefore, the authors suggested that sequelae worsen over time. In our study, we observed 36 Chinese patients in 28 unrelated families, with a rate of fracture of 55.6% (20/36), lower than that in other studies. Fractures often occur under 18 years old, rather than in adulthood (≤18/>18: 37/7), which is different from studies in other countries (13). Educating patients to reduce strenuous physical exercise was a way to reduce fracture occurrence. **Table 9** shows the difference between our study and those in other countries. According to the clinical features and follow-up, ADO II appears to show a benign trend in Chinese patients.

**Table 9:** Clinical characteristics of patients with ADO II: a comparison of studies.

	No.	Fractures	Osteoarthritis^a^	Cranial nerve palsy	Osteomyelitis	Haematopoietic defects
Current study	36	55.6%	13.9%	2.8%^c^	2.8%	5.6%
Imel et al. (19)	12	100%	NA	50%^c^	16.7%	16.7%^d^
Waguespack et al. (13)	62	84%	NA	19%^c^	13%	3%
Bénichou et al. (18)	37	78%	27%^b^	16.2%	13%	NA
Bollerslev et al. (20)	15	66.7%	NA	NA	0%	NA
T. EI-Tawilet al.	29	62.1%	NA	NA	NA	NA

a patients had discomfort of joints with X-ray changes.

b hip osteoarthritis.

c visual loss.

d anemia and transfusion needed.

Patients with ADO II are at a high risk for osteoarthritis, especially in the hip joint. However, the exact reason for this high prevalence is unknown. One theory is that reduction of the elastic modulus leads to loss of shock absorbing capacity; another theory proposes deterioration of the subchondral bone vascular supply as the reason (6, 21). In our study, approximately half of the patients had discomfort of joints, but only 13.9% had osteoarthritis. Therefore, ADO II injury to articular cartilage seems to be mild.

ADO II also is associated with cranial nerve involvement, including visual loss, hearing loss and facial palsy (18). Visual loss in osteopetrosis is considered to occur in infants or children (22, 23). The reasons for visual loss include optic canal narrowing leading to optic atrophy, which is the most common pathogenesis, retinal degeneration and cerebral venous outflow obstruction leading to increased intracranial pressure, which may cause progressive visual loss (22, 23). In our study, only one patient became blind after birth and diagnosed with optic atrophy. In addition, none of the patients had visual deterioration or loss over time. It seems that visual loss originates from childhood and is less likely to affect adults. The reason why children are vulnerable to blindness is unclear, but some studies have suggested that bone remodelling of the optic canal calibre in childhood is related to growth of the optic nerve (13).

For ADOII patients, jaw osteomyelitis is difficult to heal and may be related to ineffective osteoclastic resorption and reduced vascularity of bone (24, 25). Bollerslev thought that dental care would help to prevent jaw osteomyelitis (4), though Bénichou reported that four patients with osteomyelitis had satisfactory dental hygiene (26). Some reports (25, 27) have considered that tooth extraction was a key factor in osteomyelitis. Compared with studies abroad, only one patient (2.8%), with a low incidence, had jaw osteomyelitis in our study.

Bone marrow failure often occurs in autosomal recessive osteopetrosis (ARO) and leads to anemia, thrombocytopaenia, recurrent infection and hepatosplenomegaly, usually in infants (28). There are some reports (29, 30) of ADO II patients with haematological defects, and Ianr Walpol et al. (29) suggested that this occurs because of disease heterogeneity. In our study, only two patients (P17 and P20) exhibited the characteristics of bone marrow failure, such as anemia, thrombocytopaenia and splenomegaly. Some patients in our study had hypochromic microcytic anemia and we confirmed that one of them (P36) was an iron-deficiency anemia. As for other patients, we will continue follow-up the change of complete blood counts.

BMD measurement plays an important role in assessing fracture risk in the general population, but some studies have reported that increased BMD does not reflect bone quantity or risk of fracture (31, 32). Sebastian Butscheidt et al. suggested that DXA Z score is higher than 6.0 may be indicative for an inheritable high bone mass disorder like ADO II (33). In our study, the Z score of lumbar spine in 30 patients (30/31) is higher than 6. During follow-up, we found BMD measurements to be stable in most patients. However, the BMD value of P20 increased by 9 SD at lumbar vertebrae 1-4 over 12 years, and she experienced from haematological defects. Therefore, the change in BMD may partly reflects progression of the disease.

The most common *CLCN7* mutation in our study was p.Arg767Trp (16.2%), followed by p.(Tyr99Cys) (10.8%), p.(Arg286Gln) (10.8%), and p.(Glu313Lys) (8.1%), different from a study abroad in which c.643G>A (p.Gly215Arg) and c.856C>T [p.(Arg286Trp)]were the most common mutations (13, 34, 35). The p.Gly215Arg mutation appeared to be responsible for not only severe fractures but also visual loss, osteomyelitis and bone marrow failure in one study (13). According to our previous study (8), p.Arg767Trp is a common mutation in the Chinese population. It has been reported that patients with mutation p.(Tyr99Cys) have mild symptoms such as mild anemia, with multiple fractures in only one case (36). Similar to that report (36), there were no obvious symptoms except multiple fractures in two patients among the four present patients carrying the p.(Tyr99Cys) mutation; patients harbouring the p.(Glu313Lys) mutation had severe clinical manifestations, such as visual loss, osteomyelitis and even bone marrow failure. Therefore, we suggest that p.(Glu313Lys) may be related to the severity of clinical manifestations in ADO II. T Schmidt-Rose et al. proposed a hypothesis that mutations located in intramembrane α-helices, such as p.(Tyr99Cys), p.(Arg286Gln) and p.(Glu313Lys), create a positive electrical potential site and might impede the fast diffusional transit of Cl^−^, which may explain the less severe phenotype observed in patients with heterozygous mutations in these helices (37, 38). However, as patients with the p.(Glu313Lys) mutation had severe manifestations, it seems that it affects the functions of CLC-7 through another mechanism. The p.(Tyr99Cys) mutant disrupts interaction between the N-terminus and the transmembrane domain (TMD), thus affecting stability between the CBS domain and TMD and transporter function (39). This may be another reason why patients with p.(Tyr99Cys) mutation display mild manifestations.

There were some limitations in our study. First, it involved a relatively small sample, and only affected patients were examined. Therefore, we were unable to explore whether unaffected carriers might become affected patients over time. Second, the reason why p.(Glu313Lys) mutation, which is located in an intramembrane α-helix, caused severe manifestations in our patients is unclear. Nevertheless, we report some new findings. Bone fragility fractures often occur under 18 years old, which is different from findings in other countries. ADO II seems a benign disorder over time. Most patients in our study even showed partial improvement, including fewer fractures and alleviated joint pain. Moreover, we found some relationships between phenotype and genotype; for example, p.(Glu313Lys) mutation cause severe symptoms.

## Conclusion

This study is the largest Chinese cohort and summarizes the clinical manifestations and auxiliary examinations of 36 Chinese ADO II patients, with follow-up for some of them. We found that fractures usually occurred in childhood and that visual loss and osteomyelitis were rare. ADO II seems to have a benign course over time in Chinese patients. The mutation p.Arg767Trp is the most common mutation in the Chinese population, whereas the mutation p.(Glu313Lys) seems related to severe phenotype.

## Data Availability Statement

The datasets presented in this study can be found in online repositories. The names of the repository/repositories and accession number(s) can be found in the article/supplementary material.

## Ethics Statement

The studies involving human participants were reviewed and approved by Ethics Committee of Shanghai Jiao Tong University Affiliated Sixth People’s Hospital. Written informed consent to participate in this study was provided by the participants’ legal guardian/next of kin. Written informed consent was obtained from the individual(s) for the publication of any potentially identifiable images or data included in this article.

## Author Contributions

ZW and XL designed the report. ZW drafted the manuscript. YW were responsible for BMD examination. WF and YL were responsible for DNA extracting. ZZ and CW were responsible for the critical revision of the manuscript. All authors contributed to the article and approved the submitted version.

## Funding

This study was supported by the National Key R&D Program of China (2018YFA0800801) and the National Natural Science Foundation of China (81770871 and 81770872).

## Conflict of Interest

The authors declare that the research was conducted in the absence of any commercial or financial relationships that could be construed as a potential conflict of interest.

## Publisher’s Note

All claims expressed in this article are solely those of the authors and do not necessarily represent those of their affiliated organizations, or those of the publisher, the editors and the reviewers. Any product that may be evaluated in this article, or claim that may be made by its manufacturer, is not guaranteed or endorsed by the publisher.
